# A New Product of Bilirubin Degradation by H_2_O_2_ and Its Formation in Activated Neutrophils and in an Inflammatory Mouse Model

**DOI:** 10.3390/biom12091237

**Published:** 2022-09-04

**Authors:** Fei-Fei Yu, Yao Yuan, Yan Ao, Li Hua, Wu Wang, Yiyi Cao, Jing Xi, Yang Luan, Shangwei Hou, Xin-Yu Zhang

**Affiliations:** 1 Institute of Environmental Pollution and Health, School of Environmental and Chemical Engineering, Shanghai University, Shanghai 200444, China; 2 Shanghai Jiao Tong University-Hangzhou Future Sci-Tech City Joint Research Center for Tumor Immunotherapy, Hangzhou Innovation Institute for Systems Oncology, Hangzhou 311121, China; 3 Ministry of Education-Shanghai Key Laboratory of Children’s Environmental Health, Xinhua Hospital, Shanghai Jiao Tong University School of Medicine, Shanghai 200092, China; 4 School of Public Health, Hongqiao International Institute of Medicine, Shanghai Jiao Tong University School of Medicine, Shanghai 200025, China

**Keywords:** bilirubin, hydrogen peroxide, degradation, neutrophil, inflammation, hemorrhage

## Abstract

Bilirubin (BR) is a tetrapyrrolic compound stemming from heme catabolism with diverse physiological functions. It can be oxidized by H_2_O_2_ to form several degradation products, some of which have been detected in vivo and may contribute to the pathogenesis of certain diseases. However, the oxidative degradation of BR is complex and the conditions that BR degradation occurs pathophysiologically remain obscure. Neutrophils are known to generate large amounts of reactive oxygen species, including H_2_O_2_, upon activation and they are mobilized to inflammatory sites; therefore, we hypothesized that activated neutrophils could cause BR degradation, which could occur at inflammatory sites. In the present study, we investigated BR degradation by H_2_O_2_ and identified hematinic acid (BHP1) and a new product BHP2, whose structure was characterized as 2,5-diformyl-4-methyl-1*H*-pyrrole-3-propanoic acid. An LC-MS/MS method for the quantitation of the two compounds was then established. Using the LC-MS/MS method, we observed the concentration-dependent formation of BHP1 and BHP2 in mouse neutrophils incubated with 10 and 30 μM of BR with the yields being 16 ± 3.2 and 31 ± 5.9 pmol/10^6^ cells for BHP1, and 25 ± 4.4 and 71 ± 26 pmol/10^6^ cells for BHP2, respectively. After adding phorbol 12-myristate 13-acetate, a neutrophil agonist, to 30 μM of BR-treated cells, the BHP1 yield increased to 43 ± 6.6 pmol/10^6^ cells, whereas the BHP2 one decreased to 47 ± 9.2 pmol/10^6^ cells. The two products were also detected in hemorrhagic skins of mice with dermal inflammation and hemorrhage at levels of 4.5 ± 1.9 and 0.18 ± 0.10 nmol/g tissue, respectively, which were significantly higher than those in the non-hemorrhagic skins. BHP2 was neurotoxic starting at 0.10 μM but BHP1 was not, as assessed using *Caenorhabditis elegans* as the animal model. Neutrophil-mediated BR degradation may be a universally pathophysiological process in inflammation and can be particularly important under pathological conditions concerning hemorrhage.

## 1. Introduction

Bilirubin (BR) is the terminal product of mammalian heme catabolism [1,2]. It is a tetrapyrrolic compound and has long been considered only to be a waste product associated with liver diseases. However, mounting evidence suggests that BR is an important physiological antioxidant and has diverse biological functions, and may even be a metabolic hormone [2,3,4,5,6,7]. It may play an important role in human health and disease [2,3,5,7,8].

**Scheme 1:**
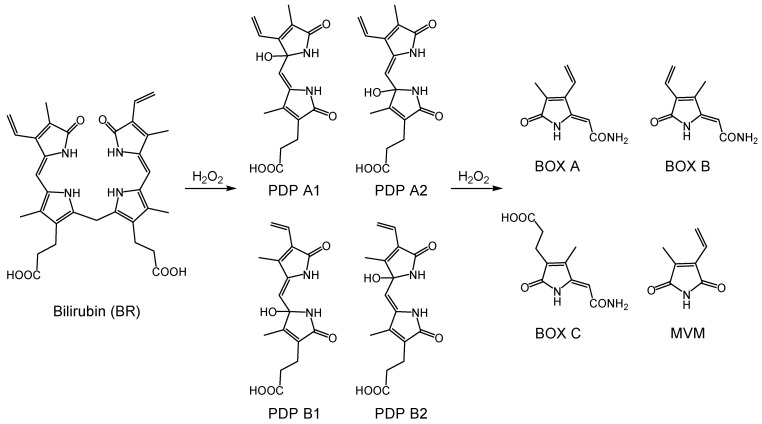
The structures of BR and its degradation products by H_2_O_2_ as reported in the literature [9,10,11] (PDPs, propentdyopents; MVM, 4-methyl-3-vinylmaleimide; BOX, bilirubin oxidation end product).

As an antioxidant, BR can readily be oxidized by reactive oxygen species (ROS) to degrade into smaller molecules, typically monopyrrolic and bipyrrolic products. It has been reported that BR can be degraded by H_2_O_2_ into several products, including 2-(3-ethenyl-1,5-dihydro-4-methyl-5-oxo-2*H*-pyrrol-2-ylidene)acetamide (bilirubin oxidation end product (BOX) A), 2-(4-ethenyl-1,5-dihydro-3-methyl-5-oxo-2*H*-pyrrol-2-ylidene)acetamide (BOX B), 5-(2-amino-2-oxoethylidene)-2,5-dihydro-4-methyl-2-oxo-1*H*-pyrrole-3-propanoic acid (BOX C), 4-methyl-3-vinylmaleimide (MVM), and four isomeric bipyrrolic products called propentdyopent (PDP) A1, A2, B1, and B2 (which were totally referred to as PDPs) (Scheme 1) [9,10,11]. Another study showed that BR degradation by H_2_O_2_ in the presence of iron formed more than 10 products with unknown structures [12].

Given the importance of BR as a physiological antioxidant with various biological functions [2,3,4,5,6,7,8], whether BR degradation occurs in vivo in significant amounts and what roles the degradation products may play in human health and diseases are apparently critical issues. A few laboratories have investigated the issues and confirmed the in vivo presence of several degradation products, including BOX A, B, and C, and PDPs [10,13,14,15,16,17,18]. Further, BOX A and B (totally referred to as BOXes) and PDPs exhibit multiple adverse effects on cells and tissues, and may be involved in the pathology of two diseases, subarachnoid hemorrhage (SAH) and cholestatic liver failure [13,14,17,19,20,21,22,23].

However, in all the studies, the degradation products were isolated from the reaction mixtures of BR with H_2_O_2_ and were characterized, and then were directly detected in biological samples, including blood, bile, gallstones, and cerebrospinal fluid (CSF) [9,10,11,13,16,17,18]. The in vivo formation of the products has been attributed to the strong oxidative environment under pathological conditions [9,13,14,18]; however, the specifically pathological conditions remain unclear. Moreover, as pointed out by Ritter et al., many oxidative degradation products of BR have actually not been identified yet [10].

In the present study, we set out to address these issues. Because neutrophils are known to be mobilized to inflammatory sites and rapidly generate large amounts of ROS (including H_2_O_2_) upon activation via a process called respiratory burst [24,25], we hypothesized that activated neutrophils could degrade BR, which would occur under inflammatory conditions. Thus, we first investigated BR degradation by H_2_O_2_ in an attempt to search for new products, subsequently established an LC-MS/MS method for the quantitation of the new products, and then examined BR degradation in mouse neutrophils using the LC-MS/MS method. The mouse neutrophils were activated by stimulation with phorbol 12-myristate 13-acetate (PMA), a common neutrophil agonist. Further, we investigated whether the BR degradation process occurred in vivo under inflammatory conditions by using a mouse model with dermal hemorrhage and inflammation, which was established through a dermal lipopolysaccharide (LPS) injection followed by a tumor necrosis factor-α (TNF-α) injection 24 h later. Lastly, we assessed the neurotoxicity of the degradation products using *Caenorhabditis elegans* as the animal model, considering that the toxicity of the degradation products may have implications for the pathophysiological consequences of BR degradation.

## 2. Materials and Methods

### 2.1. Materials

BR was purchased from TCI Shanghai (Shanghai, China). BOX A and B were obtained through synthesis as described previously [23]. Formic acid (HPLC grade) was obtained from Aladdin (Shanghai, China). Methanol (HPLC grade), acetonitrile (HPLC grade), Hank’s balanced salt solution (HBSS), and phosphate-buffered saline (PBS) were purchased from Fisher Scientific (Waltham, MA, USA). Deuterated dimethyl sulfoxide (DMSO-*d*_6_), PMA, and *Escherichia coli* LPS (O555:B5) were purchased from Sigma-Aldrich (Merck KGaA, Darmstadt, Germany). TNF-α was purchased from Sino Biological Company (Beijing, China). TRIzol^TM^ reagent was obtained from Sangon Biotech Company (Shanghai, China). Ethyl carbamate and common reagents were obtained from Sinopharm Chemical Reagent Co., Ltd. (Shanghai, China). Water was prepared by a Milli-Q Ultrapure water system (Merck KGaA, Darmstadt, Germany). NP1 was synthesized as described in our previous article [26]. Monocrotophos (MCP) was obtained from Dr. Ehrenstorfer GmbH (Augsburg, Germany). CBR00504 (2,3-dihydro-6-methyl-2-oxo-1*H*-indole-3-acetic acid, CAS No. 959241-55-5, purity 95%) was purchased from ChemBridge Corporation (San Diego, CA, USA).

A phosphate buffer (100 mM, pH 7.4) was prepared with KH_2_PO_4_ using KOH to adjust the pH. H_2_O_2_ solutions were freshly prepared prior to the experiments with the concentrations being determined spectrophotometrically at 240 nm (ε = 43.6 M^−1^cm^−1^) [27]. The stock solutions of BR (15 mM), PMA (1 mM), and NP1 (10 mM) were prepared in dimethyl sulfoxide (DMSO). The ethyl carbamate solution (5%) was prepared with PBS.

C57BL/6 mice were purchased from Shanghai Lingchang Biotechnology Co., Ltd. (Shanghai, China). The *C. elegans* strain CB7431 *bus-17(br2) X* was obtained from the Caenorhabditis Genetics Center (Minneapolis, MN, USA).

### 2.2. HPLC Analyses

HPLC analyses were carried out on an Agilent 1260 Infinity II equipped with a G7115A diode array detector (Agilent, Santa Clara, CA, USA). All HPLC analyses were performed on an Agilent ZORBAX SB-C18 column (250 × 4.6 mm, 5 μm). A linear gradient program was used, starting at 0 min from 10% to 100% pump B over 30 min (pump A, water containing 0.1% (*v*/*v*) formic acid; pump B, methanol containing 0.1% (*v*/*v*) formic acid) at a flow rate of 1.0 mL/min, maintaining 100% pump B over 2 min, at 32 min from 100% to 10% pump B over 1 min, and stopped at 34 min. The column temperature was 30 °C and the injection volume was 10 μL.

For isolation of BHP1 and BHP2, BR was reacted with H_2_O_2_ at pH 7.4 and 37 °C at the molar ratio of H_2_O_2_:BR of 30:1 for 15 h. The preparative HPLC separation was performed on a Waters SunFire C18 column (150 × 10 mm, 5 μm) (Waters Corporation, Milford, MA, USA) at a flow rate of 3.0 mL/min. The linear gradient program was the same as described above. The column temperature was 30 °C and the injection volume was 100 μL.

### 2.3. LC-MS/MS Analyses 

LC-MS/MS analyses were carried out on an Agilent 1290 Infinity HPLC system coupled with a SCIEX Triple Quad^TM^ 4500 mass analyzer (SCIEX, Framingham, MA, USA). The HPLC separation was performed on an Agilent Poroshell 120 EC-C18 column (100 × 2.1 mm, 2.7 μm). A linear gradient program was used, starting at 0 min from 10% to 100% pump B over 9 min (pump A, water containing 0.1% (*v*/*v*) formic acid; pump B, methanol containing 0.1% (*v*/*v*) formic acid) at a flow rate of 0.3 mL/min, at 11 min from 100% to 10% pump B over 0.1 min, and stopped at 11.5 min. The column temperature was 30 °C and the injection volume was 5 μL. Tandem mass spectrometric analyses were performed under the negative electrospray ionization (ESI^−^) mode with the settings being as follows: ion spray voltage (ISV), −4500 V; ion source heater temperature (TEM), 500 °C; ion source gas 1 (GS1), 50 psi; ion source gas 2 (GS2), 50 psi; curtain gas (CUR), 35 psi; collision gas (CAD), 8 psi. The analytes were detected under the multiple reaction monitoring (MRM) mode.

For quantitation, CBR00504 was added to the samples and equal amounts of CBR00504 were added to the water with volumes identical to the samples. The ion suppression ratios were calculated by dividing the peak areas of CBR00504 in the samples by those in the water. The amounts of BHP1 and BHP2 in the samples were determined on the basis of their peak areas after compensation using the ion suppression ratios.

### 2.4. Mass and Nuclear Magnetic Resonance (NMR) Spectra 

High-resolution ESI^−^ mass spectra were obtained on an LC-Q-TOF-MS/MS system, which was composed of an Agilent 1290 Infinity HPLC system coupled with a Sciex Triple TOF 4600 mass spectrometer. The chromatographic separation was accomplished on an Agilent Extent C18 (50 × 2.1 mm, 1.8 μm) column at 30 °C. A linear gradient program was used, starting at 0 min from 10% pump B and maintaining 10% pump B over 1 min (pump A, water containing 0.1% (*v*/*v*) formic acid; pump B, acetonitrile) at a flow rate of 0.3 mL/min, at 1 min from 10% pump B to 95% pump B over 5 min, maintaining 95% pump B over 3 min, at 9 min from 95% pump B to 10% pump B over 0.1 min, and stopped at 12 min. The injection volume was 1 μL. The tandem mass spectrometric analysis was performed under the ESI^−^ mode with the settings as follows: ISV, −5000 V; TEM, 500 °C; CUR, 35 psi; GS1, 50 psi; GS2, 50 psi; declustering potential (DP) 100 V; collision energy (CE), 5 V.

NMR data were collected on a Bruker Instruments Avance AV500 spectrometer (500 MHz) (Bruker Corporation, Billerica, MA, USA).

### 2.5. Reactions of BR with H_2_O_2_ under In Vitro Physiological Conditions 

A BR stock solution was prepared by dissolving 3.0 mg (5.1 μmol) of BR in 80 μL of 0.15 M NaOH. After being fully dissolved, 15 μL of the BR solution (1.0 μmol) was added to 5 mL of the 100 mM phosphate buffer (pH 7.4), to which 50 μL of the 0.1 M H_2_O_2_ solution was added. The concentration of BR and H_2_O_2_ in the reaction mixture was 0.20 and 1.0 mM, respectively. The reaction mixture was protected from light and was incubated at 37 °C. Aliquots were withdrawn every hour for HPLC and/or LC-MS/MS analyses. The reactions were also performed at the H_2_O_2_:BR molar ratio of 10:1 and 20:1 by increasing the H_2_O_2_ amounts.

### 2.6. BR Incubation with Neutrophils 

As a type of immune cells, neutrophils are sensitive to microorganisms. Therefore, all experiments using neutrophils were carefully performed under sterile conditions until the last steps for measurements (cell lysis or fluorescence detection).

Murine neutrophils were isolated as described in the literature [28,29]. BR solutions in DMSO (5 or 15 mM, 2 μL) were added to 1 mL of freshly prepared neutrophil suspensions (2 × 10^6^ cells/mL), which were incubated for 1 h in a cell culture chamber at 37 °C under 5% CO_2_. PMA solutions in DMSO (0.5 mM, 2 μL) were added and the neutrophil suspensions were incubated for another 2 h. For controls, equal volumes of DMSO were added. After sonication, 9 mL of cold anhydrous ethanol was added and the mixtures were placed at −20 °C for 30 min. Then, the mixtures were centrifuged at 4700× *g* for 20 min at 4 °C and the supernatants were concentrated to dryness in a Thermo Scientific Savant™ SPD131DDA Speedvac™ vacuum concentrator (ThermoFisher Scientific, Waltham, MA, USA). The residues were resolubilized with 50 μL of water and then were subject to LC-MS/MS analyses. The injection volumes were 8 μL.

### 2.7. Intracellular H_2_O_2_ Detection in Neutrophils by Fluorescence Probe NP1

Freshly prepared neutrophil suspensions were incubated with 5 μM NP1 in a cell culture chamber at 37 °C under 5% CO_2_ for 30 min. The cells were washed twice with HBSS and then suspended in HBSS at 1 × 10^6^ cells/mL. The neutrophil suspensions were added to plates and then BR or H_2_O_2_ solutions were added at a final concentration of 10 μM or 500 μM, respectively. After incubation at 37 °C under 5% CO_2_ for 1 h, the PMA solution was added at a final concentration of 1 μM and incubation was continued for 30 min. The fluorescence intensities were recorded on a BioTek plate reader (Agilent, Santa Clara, CA, USA) (λ_ex_ = 405 nm, λ_em_ = 555 nm, 37 °C).

### 2.8. Animal Experiments 

All animal experiments were carried out in accordance with the Guide for the Care and Use of Laboratory Animals as recommended by the U.S. National Institute of Health, and were approved by the Scientific Investigation Board of Shanghai Jiao Tong University School of Medicine, the Department of Lab Animal Science, and the Animal Care and Welfare Committee of Shanghai Jiao Tong University School of Medicine. C57BL/6 mice were maintained in specific pathogen-free rooms with controlled temperature (20–26 °C), humidity (30–70%), and lighting (12 h light-dark cycle), and had access to food and water ad libitum.

The animal experiment was carried out with 9 male mice (6–8 weeks, 18–22 g). The mouse model with inflammation and hemorrhage was established following the experimental procedure as described by Qian et al. with minor modifications [28]. Briefly, mice were anaesthetized by an intraperitoneal injection of 100 μL of 5% ethyl carbamate. The dorsal skins were shaved and LPS solutions (80 μg in 80 μL of PBS) were injected in the right dorsa with a Hamilton syringe and a 22 G needle. For negative controls, 80 μL of sterile PBS was injected in the left dorsa. After 24 h, TNF-α solutions (0.2 μg) or PBS in the same volume were injected into the same sites that received LPS or PBS, respectively. The mice were sacrificed 24 h later. For each mouse, the hemorrhagic skin was excised first and then the skin at the control site with an identical area was excised. The excised skins were weighed and then were lysed with TRIzol^TM^ reagent (1 mL of TRIzol^TM^ per 0.1 g of tissue). After homogenization with an automatic tissue grinder (60 Hz, 10 min), 9 volumes of anhydrous ethanol were added and the mixtures were centrifuged at 4700× *g* at 4 °C for 20 min. The supernatants were concentrated to dryness, then 1 mL of water and 9 mL of anhydrous ethanol were added, and the suspensions were centrifuged again at 4700× *g* at 4 °C for 10 min. The solvents were removed, and the residues were resolubilized with 100 μL of methanol and were then subjected to LC-MS/MS analyses. The injection volumes were 5 μL.

### 2.9. C. elegans Strain Maintenance and Experiments

The *C. elegans* strain CB7431 *bus-17(br2) X*, a surface-altered and drug-sensitive strain [30], was used in the present study. Worms were maintained on a nematode growth medium (NGM, containing 17 g/L Agar B, 51 mM NaCl, 2.5 g/L peptone, 25 mM pH 6.0 phosphate buffer, 1 mM MgSO_4_, 1 mM CaCl_2_, and 5 mg/L cholesterol), which were seeded with *Escherichia coli* strain OP50 as the food source. A 1% NaClO solution in 0.5 mM NaOH was used to obtain synchronized nematode populations. The gravid hermaphrodites were lysed in the solution to release eggs. The eggs hatched overnight at 20 °C without food and the hatched L1 stage larvae were transferred to NGM plates seeded with *E. coli* OP50. After 48 h, the L4 age-synchronized worms were harvested for the experiments. 

Nematodes were exposed to different concentrations of BHP1 and BHP2 in the presence of the food source for 24 h. Then, the nematodes were dispensed onto NGM plates without bacteria. After adaptation for 30 s, the frequencies of head thrash, measured by the numbers of head thrashes in 1 min, were determined. One head swing was defined as the swinging of the nematode head from one side to the other and then back again. Fifteen or thirty nematodes in each group were measured. 

### 2.10. Statistical Analysis 

Values with normal distribution are expressed as means ± standard deviations (SD). For variables with normal distribution, the independent Student’s *t* test was utilized to compare the difference between two groups, and differences among three or more groups were analyzed by a one-way analysis of variance (ANOVA), followed by a Bonferroni multiple comparison posttest. Statistical analyses were performed with SPSS version 22.0 (SPSS, Inc., Chicago, IL, USA). A *p* value < 0.05 was considered statistically significant.

## 3. Results

### 3.1. Degradation of BR by H_2_O_2_ Generated BOXes, PDPs, and a Few Additional Products 

To search for new products, BR degradation by H_2_O_2_ was investigated at in vitro physiological conditions (pH 7.4, 37 °C) with the BR and H_2_O_2_ concentrations being 200 and 1000 μM, respectively. Such concentrations are achievable in vivo under pathological conditions; BR concentrations in jaundiced infants can exceed 300 μM [2] and H_2_O_2_ concentrations in the phagosomes of activated neutrophils are estimated to be in the low micromolar range [31]. It needs to be noted that BR solubility is very low at pH 7.4 and 37 °C (~7 nM) but increases steeply with increasing pH values [32]. Furthermore, it has been shown that supersaturated BR does not precipitate readily from solutions [33]; therefore, the reaction mixture was prepared by dissolving BR in 150 mM NaOH and then diluted by >300-fold with a pH 7.4 phosphate buffer (see Section 2 for details). 

**Figure 1:**
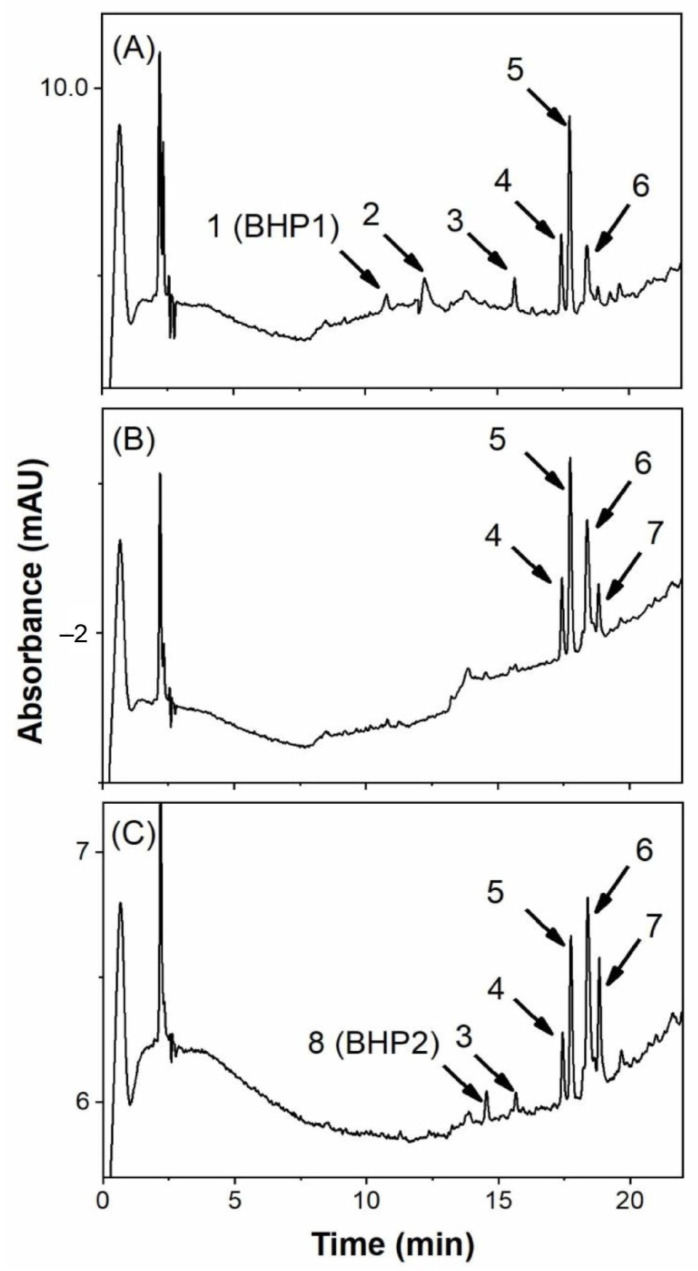
Typical HPLC chromatograms of the reaction mixtures of BR (200 μM) and H_2_O_2_ (1.0 mM) under in vitro physiological conditions (pH 7.4, 37 °C) for 48 h. The chromatogram was presented at (**A**) 260, (**B**) 280, and (**C**) 300 nm.

The reaction of BR with H_2_O_2_ proceeded slowly. It took several hours for tiny peaks to be observed on HPLC-UV. Figure 1A shows a typical chromatogram of the reaction mixture at 48 h with the monitoring wavelength at 260 nm, in which six peaks at 10.6, 12.3, 15.6, 17.4, 17.7, and 18.4 min (peaks 1–6) can be observed. The chromatogram at different wavelengths exhibited different peak profiles. At 280 nm, only peaks 4–6 and an additional peak at 18.8 min (peak 7) were visible (Figure 1B); at 300 nm, peaks 3–7, together with a new peak at 14.5 min (peak 8) were observed (Figure 1C). However, none of these peaks were the signals of BOX A and B because their retention times determined with the standards, which were 15.1 and 15.8 min, respectively, did not match those of any peaks.

Nonetheless, BOX A and B were indeed formed in the reaction but could be detected only by using LC-MS/MS under the highly sensitive MRM mode, indicating that their yields were extremely low. BOX C was not examined due to a lack of the standard. On the other hand, peaks 5, 6, and 7 were identified as the signals of PDPs based on a comparison of their UV absorption and mass spectra with those reported in the literature [11].

Therefore, peaks 1, 2, 3, 4, and 8 represented unidentified products. The five products were expected to have extremely low yields based on their peak areas. To minimize the workload to isolate sufficient amounts of the products for structural characterization by NMR, we used a strategy to first screen the products in biological specimens and then isolate only those that were detected with significant concentrations in the specimens. To do so, tiny amounts of the five products were isolated and loaded onto the instrument to determine the parameters for each product with the aim to establish a sensitive LC-MS/MS method under the MRM mode. However, the attempt for products 2 and 4 was unsuccessful probably due to insufficient amounts and/or low purity. Finally, an LC-MS/MS method was established for products 1, 3, and 8, and the ion transitions and instrumental parameters were listed in Table 1. The method was then used to screen the lysates of the mouse neutrophils incubated with BR (see Section 3.5), and products 1 and 8 were detected with significant signal intensities. Therefore, the two products, which were designated as BHP1 and BHP2, were isolated by preparative HPLC for structural characterization by NMR.

### 3.2. Structural Characterization of BHP1 and BHP2 

**Scheme 2:**
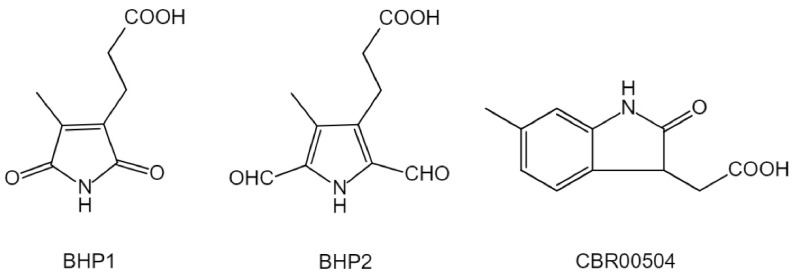
The structures of BHP1, BHP2, and CBR00504 (used as the internal standard for quantitation by LC-MS/MS).

The ESI^−^ mass spectrum of BHP1 exhibited the deprotonated molecular ion at *m*/*z* 182 and a major daughter ion at *m*/*z* 138, indicating that the product contains a carboxyl group. Based on its ^1^H NMR and heteronuclear multiple-quantum correlation (HMQC) spectra (Figures S1 and S2), and a comparison with the NMR data in the literature [34,35], BHP1 was characterized as hematinic acid (Scheme 2), a known compound. 

The high-resolution ESI^−^ mass spectrum of BHP2 exhibited the deprotonated molecular ion at *m*/*z* 208.0618 (Figure S3); thus, the molecular formula was determined as C_10_H_11_NO_4_ (the calculated *m*/*z* for the deprotonated molecular ion was 208.0610). The ^1^H NMR spectrum in DMSO-*d*_6_ (Figure S4) showed the signals of nine protons. Among them, the signals of two protons appeared at 9.829 and 9.834 ppm (Figure S5), which were on carbons with the chemical shifts at 182.7 ppm as determined by the HMQC spectrum (Figure S6). Apparently, they were the signals of two formyl groups. The spectra, together with the correlation spectroscopy (COSY) spectrum (Figure S7), also indicated the presence of a methyl group (2.27 ppm) and a -CH_2_CH_2_- moiety (2.43 and 2.92 ppm). The heteronuclear shift correlations via the multiple bond connectivity (HMBC) spectrum (Figure S8) showed that the protons on the formyl and methyl groups, and a methylene group (at 2.92 ppm), were all coupled to two carbons at 127.5 and 132.5 ppm, providing evidence that these groups were attached to a pyrrole ring. Collectively, the structure of BHP2 was characterized as 2,5-diformyl-4-methyl-1*H*-pyrrole-3-propanoic acid (Scheme 2).

### 3.3. Establishment of the LC-MS/MS Method for Quantitation of BHP1 and BHP2

With purified BHP1 and BHP2 in hand, the LC-MS/MS method was established. The parameters were listed in Table 1, and the limits of detection of BHP1 and BHP2 on the column were determined to be 0.1 and 0.05 pmol, respectively. Their working curves (Figure S9) were linear with the ranges of quantities and correlation coefficients as follows: BHP1, 0.1–300 pmol, 0.9945 (*y* = 2423.8*x*); BHP2, 0.05–200 pmol, 0.9997 (*y* = 65218*x*).

Because no isotope-labeled BHP1 and BHP2 were available, we used a commercial compound CBR00504, 2,3-dihydro-6-methyl-2-oxo-1*H*-indole-3-acetic acid (Scheme 2), as the internal standard to estimate the ion suppression effects. The compound is absent in biological samples and has structural moieties similar to BHP1 and BHP2 with a molecular weight of 205, which is very close to that of BHP2 (M.W. 209). The mass spectrometric parameters of the internal standard are listed in Table 1.

### 3.4. The Time-Dependent Formation of BHP1 and BHP2 in the Reaction of BR with H_2_O_2_

**Figure 2:**
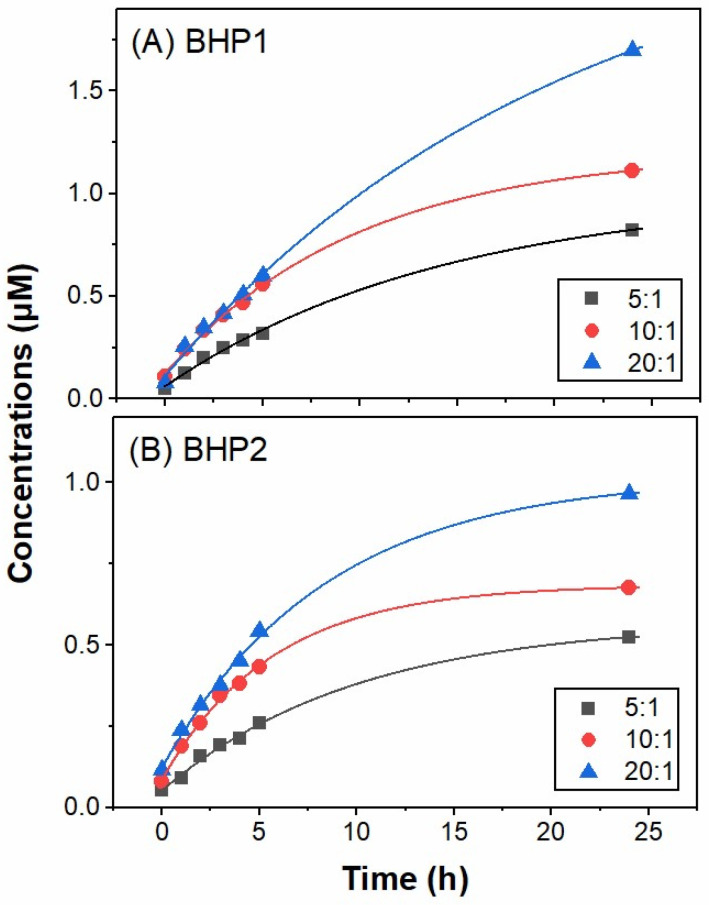
Time-dependent formation of (**A**) BHP1 and (**B**) BHP2 at different H_2_O_2_:BR molar ratios (5:1, 10:1, and 20:1). The reactions were carried in phosphate buffer (100 mM, pH 7.4) with an initial BR concentration of 200 μM and initial H_2_O_2_ concentration at 1.0, 2.0, and 4.0 mM.

Through quantitation by LC-MS/MS, the time-dependent formation of BHP1 and BHP2 was investigated. The results showed that both products were formed until 24 h, and the amounts of the two products increased with the increase in the molar ratio of H_2_O_2_:BR (Figure 2). At 24 h, the formation of BHP2 almost plateaued, whereas the amounts of BHP1 kept increasing, albeit very slowly. The yields of BHP1 and BHP2 at 24 h and the H_2_O_2_:BR molar ratio of 20:1 were 0.9% and 0.5%, respectively.

### 3.5. BR Degraded into BHP1 and BHP2 in Mouse Neutrophils 

The physiological range of serum BR concentrations is between 5 and 17 μM [5]. Thus, to investigate BR degradation by neutrophils, we set two incubation concentrations, 10 and 30 μM, which represent the physiological and pathological conditions, respectively. On the other hand, the respiratory burst of neutrophils, i.e., the rapid ROS release, is a very fast process; when PMA is used as the stimulus, the ROS release in murine neutrophils reaches the maximum at 40 min and comes to an end within 2 h [36]. Thus, we set the incubation time after PMA stimulation as 2 h.

The experiment was performed through incubating mouse neutrophils with 10 and 30 μM of BR for 1 h, and then adding PMA to the cells incubated with 30 μM BR. After incubation for 2 h, the cells were collected and lysed, and then they were subject to LC-MS/MS analyses.

**Figure 3:**
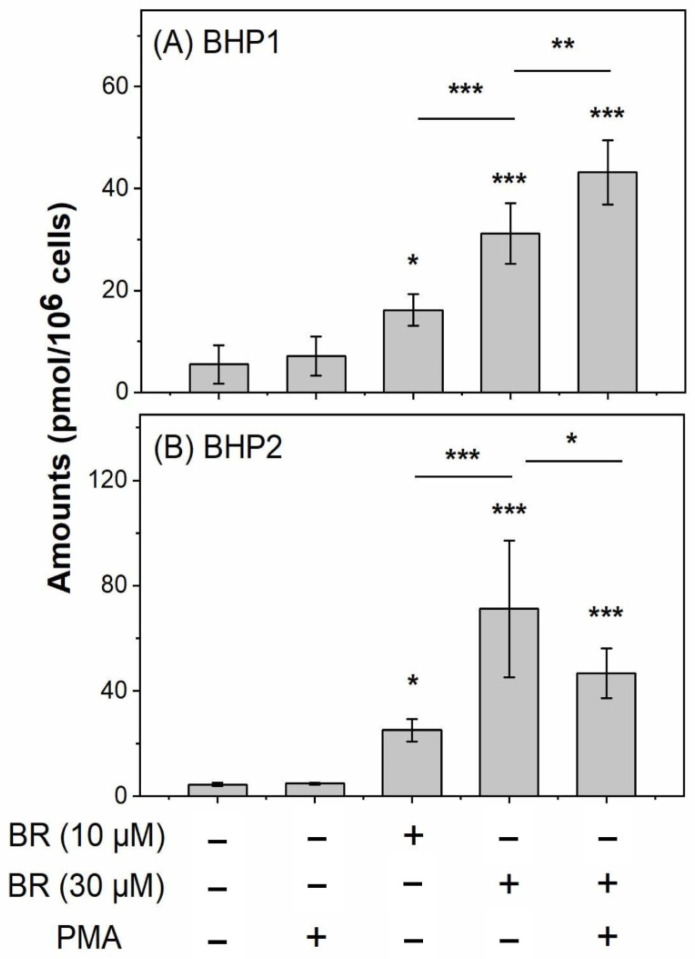
BR degradation in murine neutrophils to form (**A**) BHP1 and (**B**) BHP2. Neutrophils were incubated with BR (10 or 30 μM) for 1 h, and then PMA was added to 30 μM BR-treated cells at a final concentration of 1 μM. After incubation for 2 h, cells were lysed and subject to LC-MS/MS analyses. The data were presented as means ± standard deviation (SD) (*n* = 6 except for the negative control (*n* = 4), * *p* < 0.05, ** *p* < 0.01, *** *p* < 0.001; BR, bilirubin; PMA, phorbol-12-myristate-13-acetate).

Unexpectedly, it was observed that BR incubation caused the concentration-dependent formation of BHP1 and BHP2 without PMA stimulation (Figure 3). Specifically, upon incubation with 10 and 30 μM of BR, BHP1 was produced at 16 ± 3.2 and 31 ± 5.9 pmol/10^6^ cells, and BHP2 at 25 ± 4.4 and 71 ± 26 pmol/10^6^ cells, respectively, which were significantly greater than those in the corresponding controls. Interestingly, PMA addition induced a further increase in the amount of BHP1 but a decrease in that of BHP2 (Figure 3), and the increase or decrease was statistically significant (*p* < 0.01 or 0.05, respectively). Apparently, the results suggested that BR itself was able to activate neutrophils, which in turn caused the degradation of BR. It has been reported that BR at 4.5 μM promoted the degranulation of neutrophils but 45 μM BR had no effect [37]. The result reported in the paper suggests that BR is capable of activating neutrophils, which supports our observation, although the authors used a different endpoint (i.e., degranulation).

### 3.6. BR Stimulated Murine Neutrophils to Produce H_2_O_2_

Because the experiment in Section 3.5 suggested that BR could activate neutrophils (Figure 3), we used NP1, an H_2_O_2_-specific fluorescence probe developed in our laboratory [26], to detect the production of H_2_O_2_ in BR-incubated neutrophils. Cells incubated with 500 μM of H_2_O_2_ were used as the positive control.

**Figure 4:**
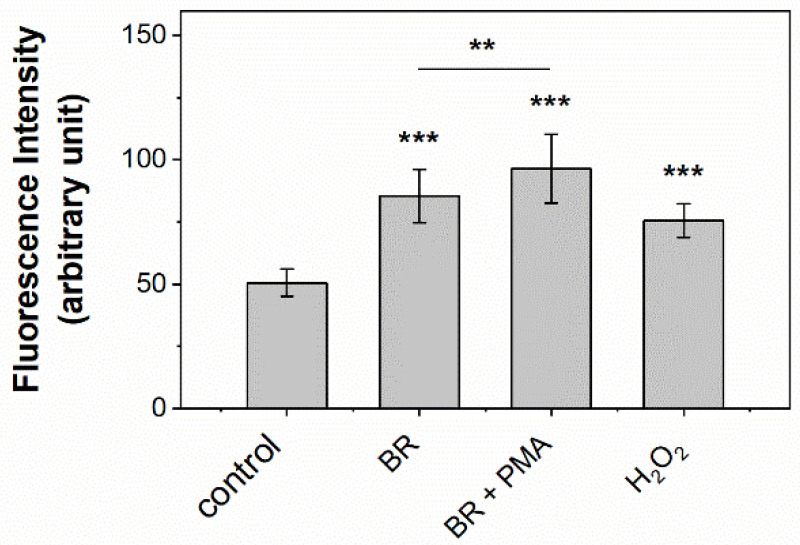
BR stimulated murine neutrophils to produce H_2_O_2_ as detected by a H_2_O_2_-specific fluorescence probe NP1 [26]. Neutrophils were incubated with NP1 (5 μM) for 0.5 h and then BR was added at a final concentration of 10 μM. After incubation for 1 h, PMA was added at a final concentration of 1 μM and incubation was continued for 0.5 h. H_2_O_2_ (500 μM) was used as the positive control. The excitation and emission wavelengths were 405 and 555 nm, respectively. The data were presented as means ± SD (*n* = 18, ** *p* < 0.01, *** *p* < 0.001; BR, bilirubin; PMA, phorbol 12-myristate 13-acetate).

The result showed that the fluorescence intensity in the neutrophils incubated with 10 μM BR was significantly higher than that in the negative control (*p* < 0.001, Figure 4). The PMA addition caused a further increase in the intensity (*p* < 0.01). Notably, both fluorescence intensities in the BR-treated and BR/PMA-treated cells were greater compared to that in the positive control, and the differences were statistically significant (*p* < 0.05 and 0.001, respectively), implying that the H_2_O_2_ concentrations in these cells may be higher than 500 μM. Therefore, the experiment showed that BR was able to stimulate murine neutrophils to generate H_2_O_2_, thus providing further evidence for the above observation that BR could activate neutrophils.

### 3.7. BHP1 and BHP2 Were Formed in Murine Skin with Inflammation and Hemorrhage 

**Figure 5:**
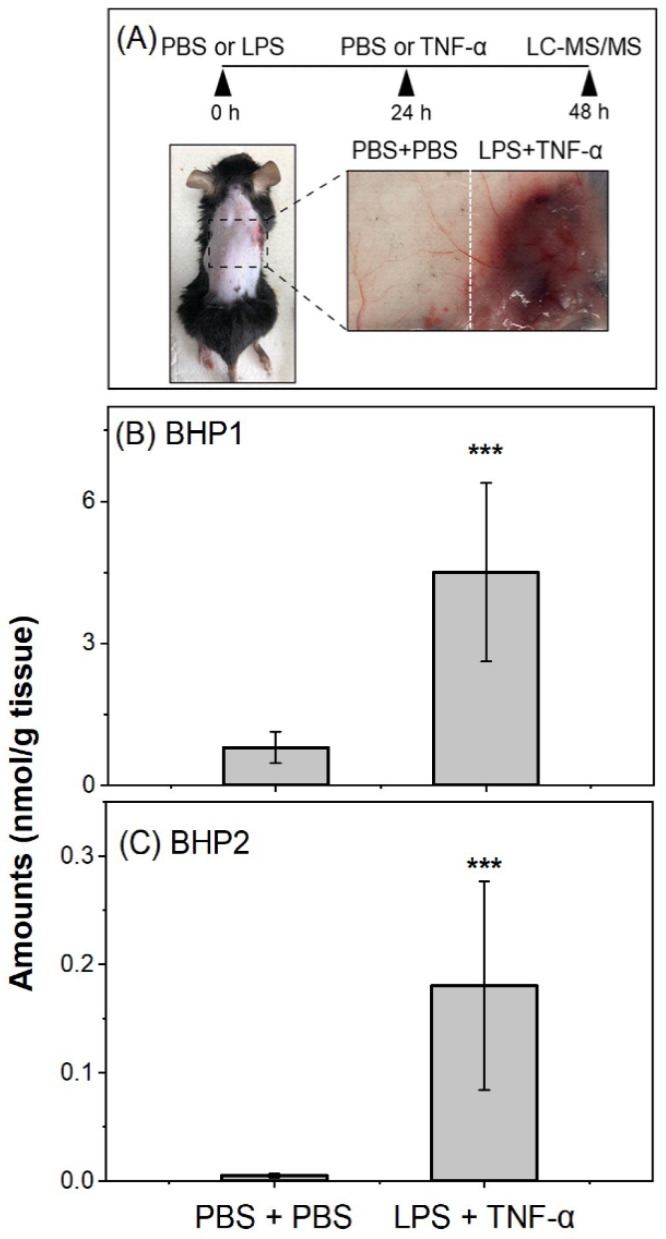
The formation of BHP1 and BHP2 in murine skin with inflammation and hemorrhage. (**A**) The mouse model was established by a dermal LPS injection followed by a TNF-α injection 24 h later to induce skin lesions with inflammation and hemorrhage. Two consecutive PBS injections were used as the control. For each mouse, the left dorsal skin was injected with PBS and the right one with LPS/TNF-α. Twenty-four hours after the TNF-α injections, skins were excised and subject to LC-MS/MS analyses after lysis by TRIzol^TM^ reagent. (**B**,**C**) The BHP1 and BHP2 levels in the skins at the control (PBS + PBS) and the lesion sites (LPS + TNF-α). The data were presented as means ± SD (*n* = 9, *** *p* < 0.001; PBS, phosphate-buffered saline; LPS, lipopolysaccharide; TNF-α, tumor necrosis factor-α).

The above experiment demonstrated that the neutrophils caused BR degradation. In the meantime, it is well known that neutrophils are mobilized to inflammatory sites in response to inflammation; consequently, one can expect that BR degradation will occur at inflammatory sites. To provide evidence for this hypothesis, we used a mouse model of a local Shwartzman reaction, as employed previously by Qian et al. [28]. In the model, a dermal LPS injection followed by a TNF-α injection 24 h later induces skin lesions resembling those of thrombo-hemorrhagic vasculitis. In effect, the skin injected with LPS and TNF-α became hemorrhagic and inflammatory, which could be easily distinguished from the surrounding skin by the naked eye (Figure 5A).

As a catabolite of heme, BR has been demonstrated to be formed at elevated levels at hemorrhagic sites [18]. Therefore, in the skin injected with LPS and TNF-α, elevated BR levels and inflammation exist at the same site, and thus BR degradation is expected to occur.

Indeed, significantly higher levels of BHP1 and BHP2 were observed in the skins with hemorrhage and inflammation as expected, compared to those in the skins at the control sites (*p* < 0.001, Figure 5B,C). Specifically, the levels of BHP1 and BHP2 in the lesional skins were 4.5 ± 1.9 and 0.18 ± 0.10 nmol/g tissue, respectively, and the values in the control skins were 0.80 ± 0.33 and 0.005 ± 0.002 nmol/g tissue, respectively (both *p* < 0.001).

### 3.8. BHP2 Exhibited Neurotoxicity in C. elegans

As reported in the literature [13,14,17,19,20,21,22,23], BOXes and PDPs have various adverse effects on cells and tissues; therefore, we assessed the neurotoxicity of BHP1 and BHP2 using *C. elegans*, a model system widely used for biomedical research and neurotoxicity assessments of environmental pollutants [38]. The endpoint of neurotoxicity was selected because BOXes and PDPs are probably involved in the pathogenesis of cerebral vasospasms after SAH [13,14]. It is likely that BR degradation products may also contribute to the pathology of intracerebral hemorrhage via neurotoxicity. In our experiment, the frequency of head thrash was used as the endpoint for neurotoxicity.

**Figure 6:**
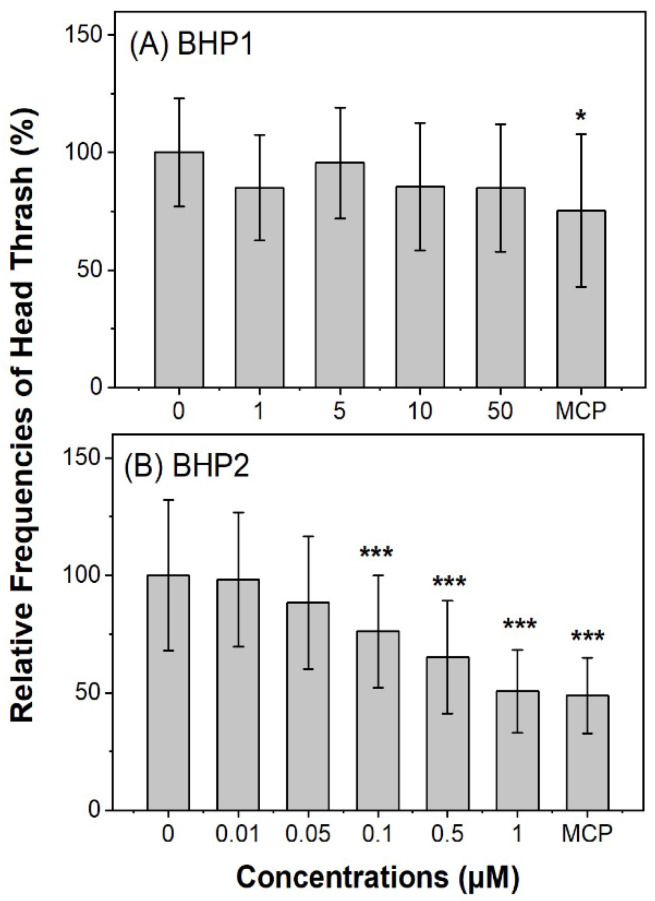
Assessment of neurotoxicity of (**A**) BHP1 and (**B**) BHP2 using *C. elegans* as the animal model with the frequency of head thrash being used as the endpoint. Monocrotophos (MCP, 75 μM for BHP1 and 100 μM for BHP2), a known neurotoxicant, was used as the positive control. The data were normalized and presented as mean ± SD (*n* = 15 and 30 for BHP1 and BHP2, respectively; * *p* < 0.05, *** *p* < 0.001).

The result showed that BHP1 did not exhibit any effect on the frequency of head thrash of *C. elegans* up to 50 μM, whereas BHP2 caused a concentration-dependent decrease in the frequency and the effect became statistically significant starting from 0.10 μM (Figure 6), suggesting that BHP2 was neurotoxic but BHP1 was not. It was noted that the highest BHP2 concentration (1 μM) in the experiment was much lower than its LC_50_ (lethal concentration, 50%) value (>75 μM).

## 4. Discussion

In the present study, our investigation on BR degradation by H_2_O_2_ led to the discovery of a new product BHP2 and hematinic acid (BHP1). We further demonstrated the formation of the two products in BR-incubated neutrophils and in murine skin with hemorrhage and inflammation, suggesting the occurrence of BR degradation ex vivo and in vivo, in particular under pathological conditions with hemorrhage and inflammation. Lastly, we found that BHP2 was neurotoxic, implicating that BR degradation may have pathological consequences.

The formation of BHP2 as a BR degradation product has not been reported previously. In fact, BHP2 is virtually a novel compound because it has not been structurally characterized before. Currently, only two pieces of literature are present in the database of the American Chemical Society. One is an article published in 2015 to screen the autoxidation products of biliverdin with LC-MS/MS, but BHP2 is only an assumed product because it was not detected at all [39]. The other is about a Japanese patent published in 2016 to claim BHP2 formation in hemin degradation by H_2_O_2_ [40]. However, it has not been certain whether the compound was actually formed because only the mass spectral data were collected; the possibility that an isomer with the same molecular weight was formed could not be excluded, particularly considering the great complexity of the degradation of BR or related compounds (protoporphyrin, hemin, biliverdin, etc.).

On the other hand, although BHP1, i.e., hematinic acid, is a known compound, its formation in BR degradation by H_2_O_2_ was not reported until 2018 [10]. It should be noted that in the paper [10], the formation of BHP1 was indicated only by an arrow in the chromatogram of the reaction mixture and there was no information about how the product was identified. Needless to say, the formation of hematinic acid in neutrophils or in vivo under inflammatory conditions has never been demonstrated before. Interestingly, hematinic acid has been extensively demonstrated to be formed in the H_2_O_2_-mediated degradation of hemoglobin, heme, ferriprotoporphyrin IX, and chlorophyll *a* [41,42,43,44], or in BR photooxidation [45,46].

Despite the fact that the yields of BHP1 and BHP2 in BR degradation by H_2_O_2_ were quite low (<1%), the yields in BR-incubated neutrophils appeared high. Based on the reported diameter of the neutrophils of C57BL/6 mice (~12 μm) [47], the intracellular concentrations of BHP1 and BHP2 are estimated to be 18 and 28 μM for 10 μM of BR-incubated neutrophils, and 34 and 78 μM for 30 μM of BR-treated neutrophils, respectively. It is worth noting that the estimated intracellular concentrations of BHP1 and BHP2 are similar or higher than the corresponding extracellular BR ones, suggesting that BR is degraded in neutrophils at quite high efficacy and that products are accumulated inside cells. The great discrepancy between the yields of the products in the reaction and in neutrophils, together with the observation that BR degradation by H_2_O_2_ is slow but the formation of the products in neutrophils is rapid, implicates that that BHP1 and BHP2 may have other sources, probably via the degradation of BR by ROS other than H_2_O_2_, given that activated neutrophils can produce a wide spectrum of ROS [24]. BR degradation by other ROS is being investigated in our laboratory.

The observation that BR is able to activate neutrophils to degrade itself is a profound finding because neutrophils are critical players in inflammatory responses [24,25,48]. It is well known that neutrophils are mobilized to inflammatory sites in response to inflammation and that, meanwhile, BR is a physiological antioxidant that is constantly present in blood; consequently, neutrophil-mediated BR degradation should be a universal pathophysiological process in inflammation. This also raises a possibility that the BR degradation products can be used as biomarkers for inflammation. The result in the mouse experiment supports the in vivo presence of BR degradation under inflammatory conditions. 

Apparently, high concentrations of BR degradation products are expected to be generated when elevated BR levels exist at inflammatory sites. Because heme or hemin itself can induce an inflammatory response and trigger the oxidative burst of neutrophils [49], and because heme is the physiological source of BR [1,2], BR degradation may be particularly important under pathological conditions with hemorrhage.

Adding to the issue is the finding that BHP2 exhibited neurotoxicity at a concentration as low as 0.10 μM. The concentration should be achievable in humans under some pathological conditions, considering that the total serum concentration of BOXes can reach 0.5 μM in patients with cholestatic liver failure and the total mean concentration of PDPs is 0.76 μM in the CSF of patients with SAH [13,17]. The finding suggests that BR degradation can have pathological consequences if sufficient amounts of degradation products are generated. It is well known that high levels of BR are neurotoxic, and apparently whether BR degradation products such as BHP2 play a role in BR neurotoxicity is an important issue that is worth investigation.

In summary, we discovered BHP2, a new product, and hematinic acid in BR degradation by H_2_O_2_. We demonstrated that neutrophils could be activated by BR to cause the formation of the two products in significant amounts. The process occurred in a mouse model with dermal hemorrhage and inflammation. Thus, the BR degradation process may be a universal pathophysiological process underlying inflammatory diseases and can be particularly important under pathological conditions with hemorrhage. BHP2 is neurotoxic, implicating that BR degradation may have pathological consequences.

## Figures and Tables

**Table 1:** The ion transitions and mass spectrometric parameters for LC-MS/MS analyses of product 1, 3, 8, and CBR00504 (the internal standard) under the MRM mode.

Compound	Retention Time (min)	Qualification or Quantification	Ion Transitions	DP ^a^ (V)	CE ^a^ (V)	EP ^a^ (V)	CXP ^a^ (V)
Product 1 (BHP1)	3.2	Qualification	182/138	−50	−14	−11	−11
		Quantification	182/123	−50	−28	−11	−11
Product 3	5.2	Qualification	369/271	−65	−21	−12	−11
		Quantification	369/289	−65	−17	−12	−11
Products 8 (BHP2)	4.4	Qualification	208/108	−51	−26	−10	−11
		Quantification	208/146	−52	−22	−10	−11
CBR00504 *	5.1	Qualification	204/158	−40	−28	−10	−11
		Quantification	204/160	−40	−18	−10	−11

^a^ DP: declustering potential; CE: collision energy; EP: entrance potential; CXP: collision cell exit potential. * internal standard.

## Data Availability

Not applicable.

## Supplementary Materials

The materials include an additional explanation about the NMR spectra of BHP2 and the structural characterization, the ^1^H NMR and HMQC spectra of BHP1, the high-resolution ESI^−^ mass spectrum and the ^1^H NMR, HMQC, COSY, and HMBC spectra of BHP2, and the standard curves of BHP1 and BHP2/. The supporting information can be downloaded at: https://www.mdpi.com/article/10.3390/biom12091237/s1.

## Abbreviations

BHP1	hematinic acid
BHP2	2,5-diformyl-4-methyl-1*H*-pyrrole-3-propanoic acid
BOX	bilirubin oxidation end product
BR	bilirubin
CAD	collision gas
CE	collision energy
COSY	correlation spectroscopy
CSF	cerebrospinal fluid
CUR	curtain gas
CXP	collision cell exit potential
DMSO	dimethyl sulfoxide
DMSO-*d*_6_	deuterated dimethyl sulfoxide
DP	declustering potential
EP	entrance potential
ESI	electrospray ionization
GS1	ion source gas 1
GS2	ion source gas 2
HBSS	Hank’s balanced salt solution
HMBC	heteronuclear multiple bond correlation
HMQC	heteronuclear multiple-quantum correlation
ISV	ion spray voltage
LPS	lipopolysaccharide
MCP	monocrotophos
MRM	multiple reaction mode
MVM	4-methyl-3-vinylmaleimide
NGM	nematode growth medium
NMR	nuclear magnetic resonance
PBS	phosphate-buffered saline
PDP	propentdyopent
PMA	phorbol-12-myristate-13-acetate
ROS	reactive oxygen species
SAH	subarachnoid hemorrhage
SD	standard deviation
TEM	ion source heater temperature
TNF-α	tumor necrosis factor-α
