# Supplementary material for: A New Product of Bilirubin Degradation by H2O2 and Its Formation in Activated Neutrophils and in an Inflammatory Mouse Model
Source: Biomolecules. 2022 Sep 4;12(9):1237. doi: 10.3390/biom12091237 (PMC9496627; doi:10.3390/biom12091237)
Supplement: Supplementary file 1 [file biomolecules-12-01237-s001.zip › biomolecules-1879852-supplementary.pdf]

Supplementary Material

A new product of bilirubin degradation by H_2O_2 and its formation in activated neutrophils and in an inflammatory mouse model

Fei-Fei Yu [1,†], Yao Yuan [2,†], Yan Ao [3], Li Hua [4], Wu Wang [1,*], Yiyi Cao [4], Jing Xi [4], Yang Luan [4], Shangwei Hou [2,*] and Xin-Yu Zhang [4,*]

Additional Explanation about the NMR spectra of BHP2 and the Structural Characterization

Initially, the isolated BHP2 sample exhibited the purity of 94% as determined by HPLC at 300 nm. Because its amount (~4 mg) was very limited and further purification could lead to a significant decrease in the amount, the NMR data, including the ^1H NMR (data not shown), HMQC (Fig. S6), COSY (Fig. S7), and HMBC spectra (Fig. S8), were collected with the BHP2 sample. In the ^1H NMR spectrum of the sample, in addition to the signals as seen in Fig. S4, 3 groups of additional peaks at ~1.3, ~6.8, and ~7.2 ppm, each of which represents 2-3 protons, were observed (see Figs. S6-S8), causing great difficulty in determining the structure. Therefore, the sample was purified again to raise the purity to ~99%. In the ^1H NMR spectrum of the 99% pure sample (Fig. S4), the peaks around 1.3, 6.8, and 7.2 ppm

seen in that of the 94% pure sample disappeared. However, the amount of the 99% pure sample was only ~1 mg, which was not sufficient for collection of the 2D NMR spectral data on a 500 MHz spectrometer. However, the 2D spectra obtained with the 94% pure sample, i.e., the HMQC, COSY, and HMBC spectra as presented in Figs. S6-S8, were able to be used to determine the BHP2 structure, once the ^{1}H NMR spectrum of pure BHP2 was successfully obtained, because the signals of the impurities did not interfere with those of BHP2 at all. Consequently, we did not collect the 2D spectral data of the 99% pure sample.

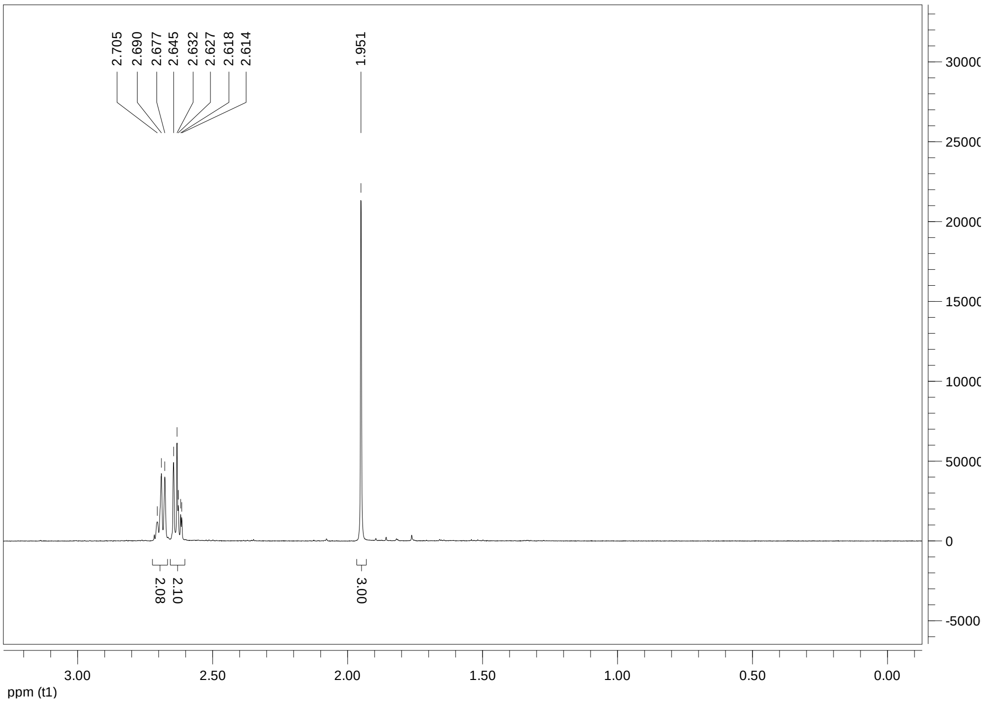

Figure S1. The ^{1}H NMR spectrum of BHP1 in D_2O (500 MHz).

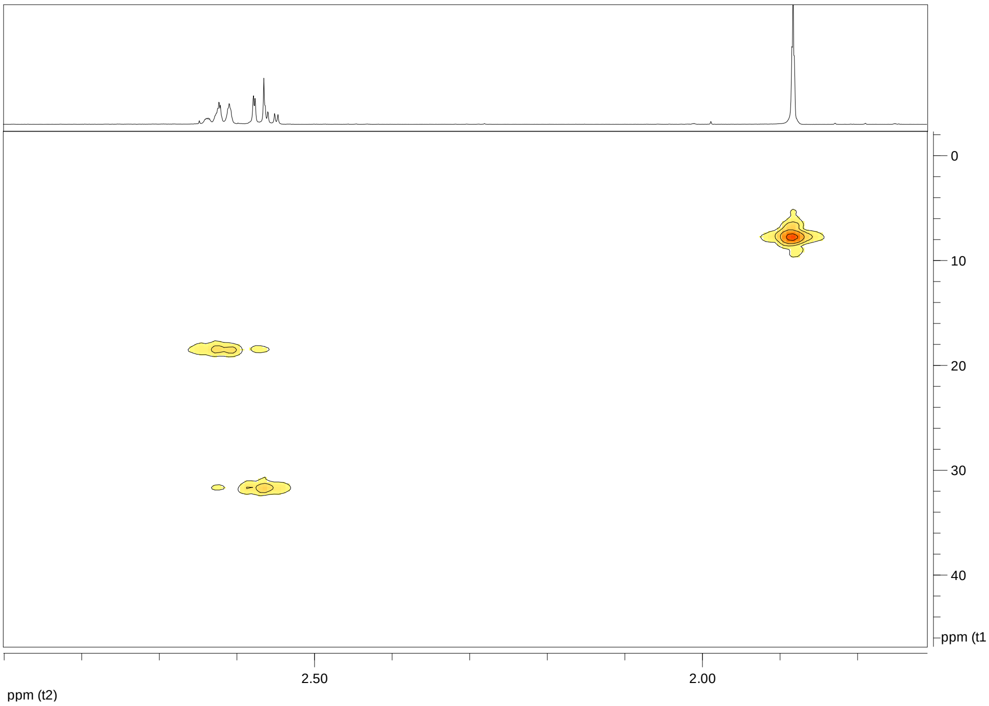

Figure S2. The HMQC spectrum of BHP1 in D_2O.

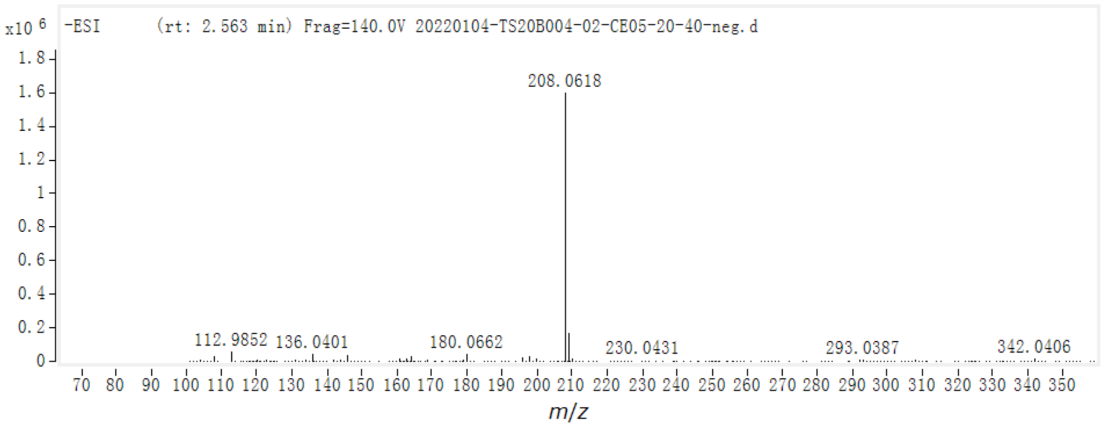

Figure S3. The high-resolution ESI⁻ mass spectrum of BHP2.

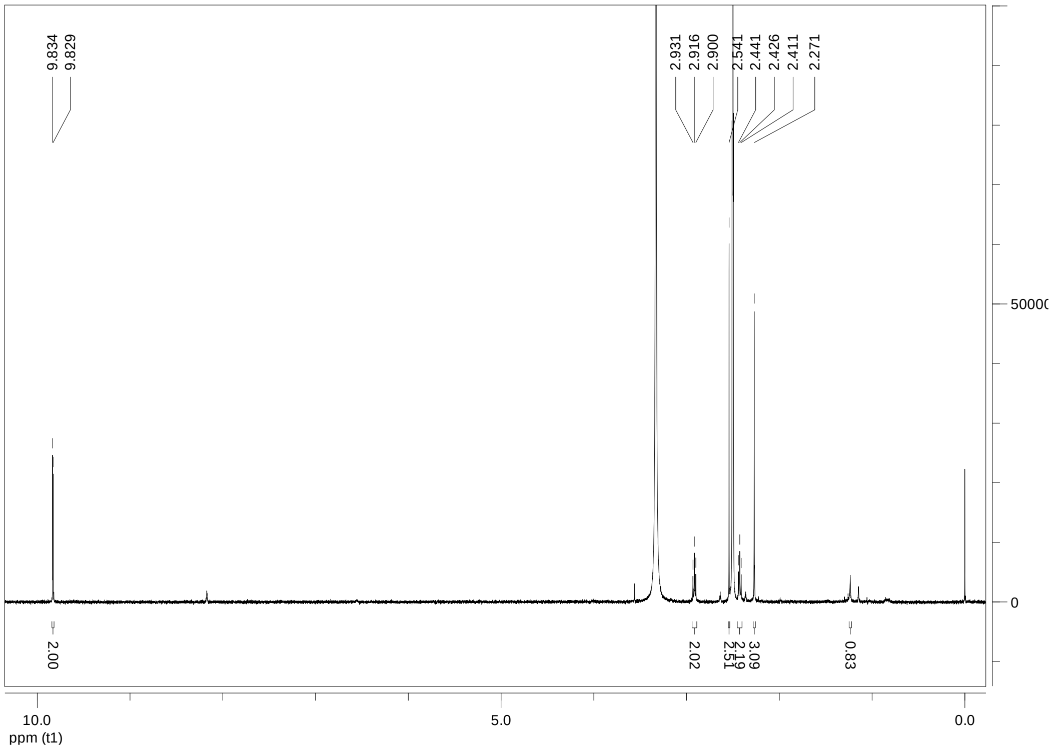

Figure S4. The ^{1}H NMR spectrum of BHP2 in DMSO-d_6 (500 MHz).

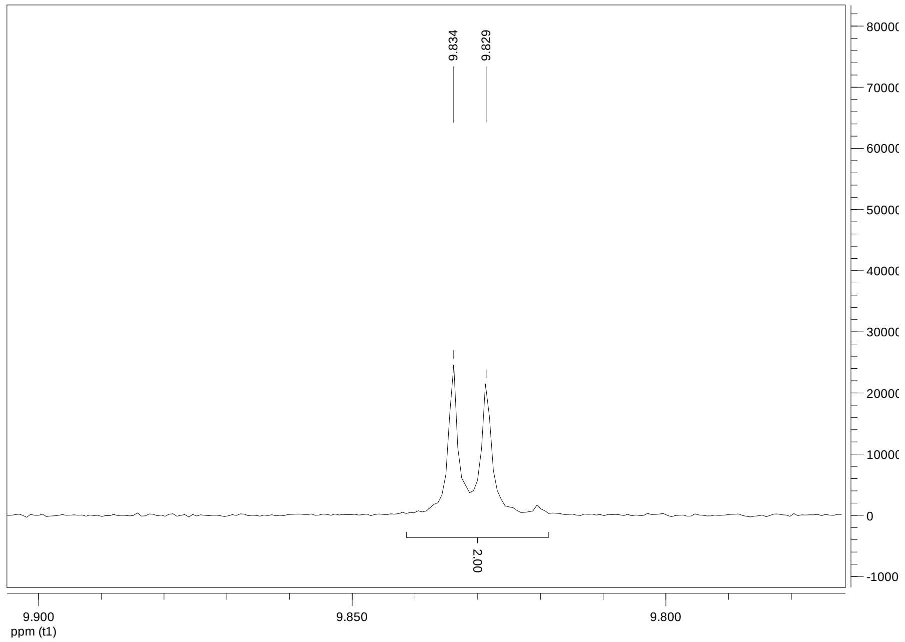

Figure S5. The ^{1}H NMR spectrum of BHP2 in DMSO-d_6 between 9.8 and 9.9 ppm to show the two single peaks at 9.829 and 9.834 ppm.

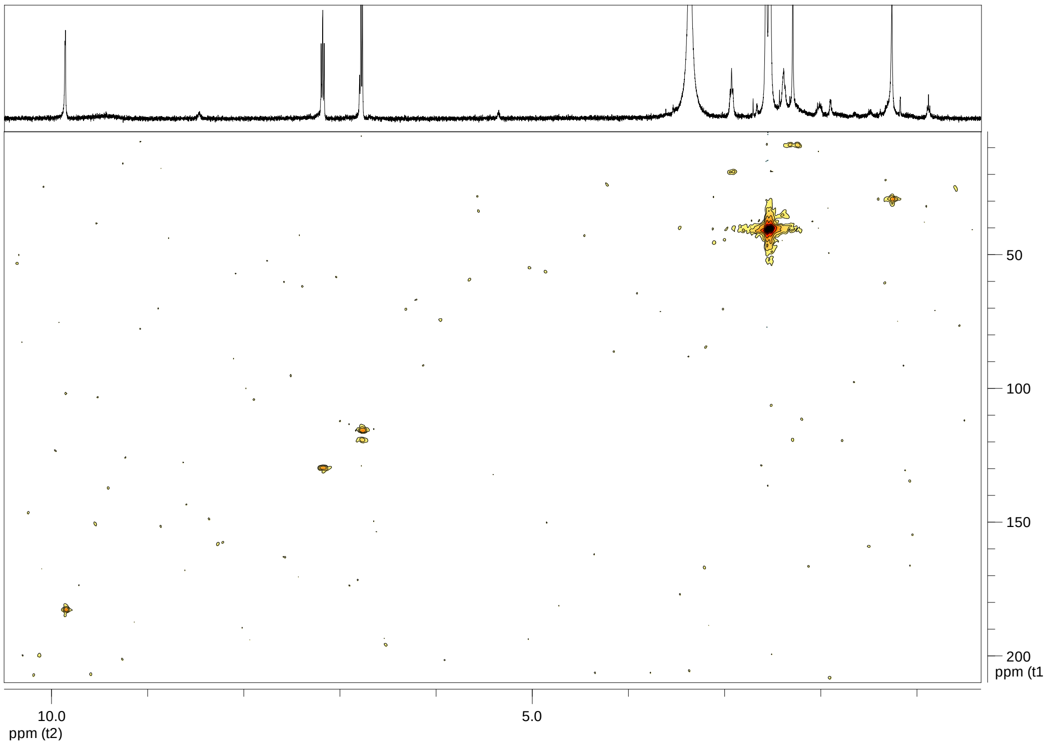

Figure S6. The HMQC spectrum of BHP2 in DMSO-d_6.

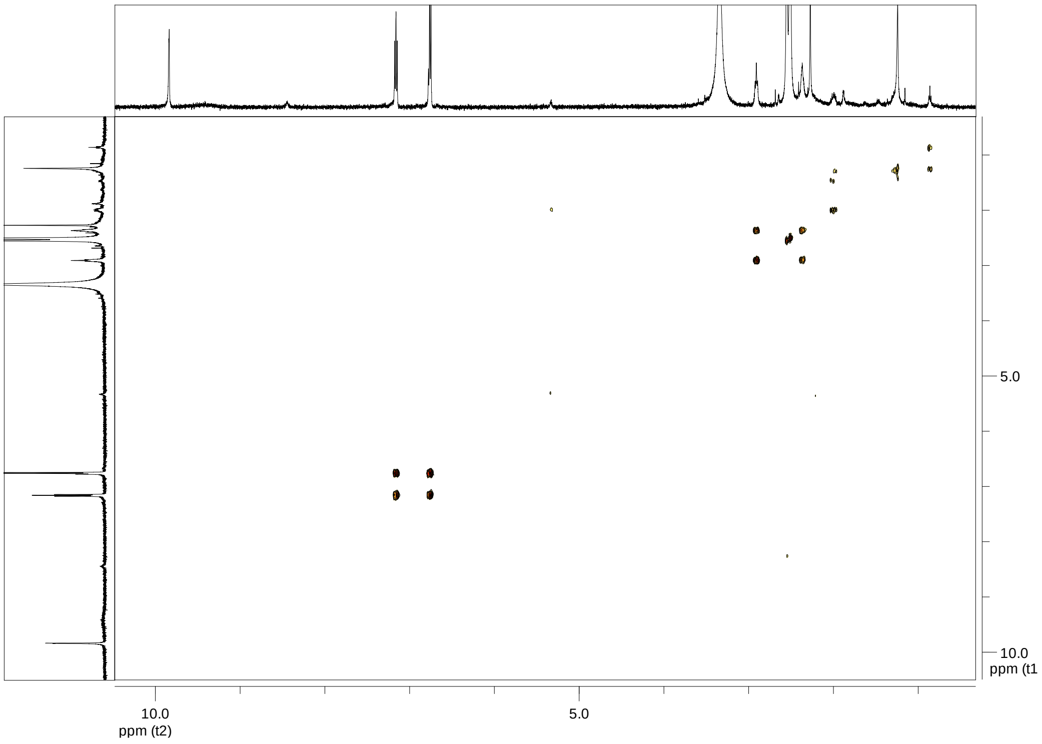

Figure S7. The COSY spectrum of BHP2 in DMSO-d_6.

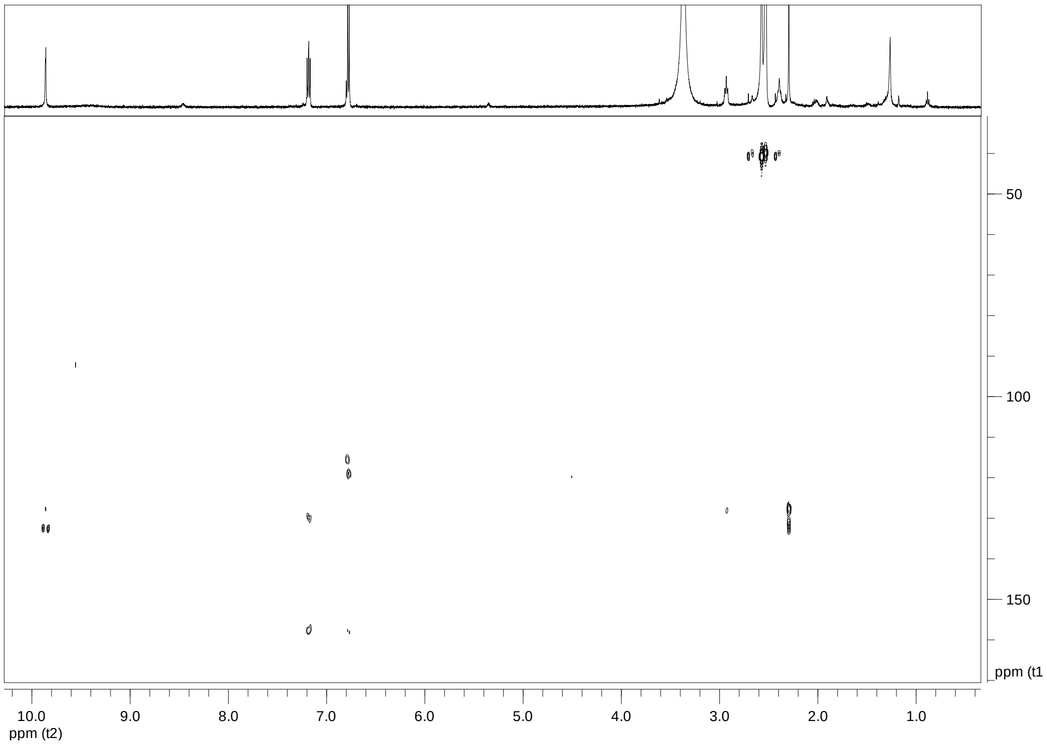

Figure S8. The HMBC spectrum of BHP2 in DMSO-d_6.

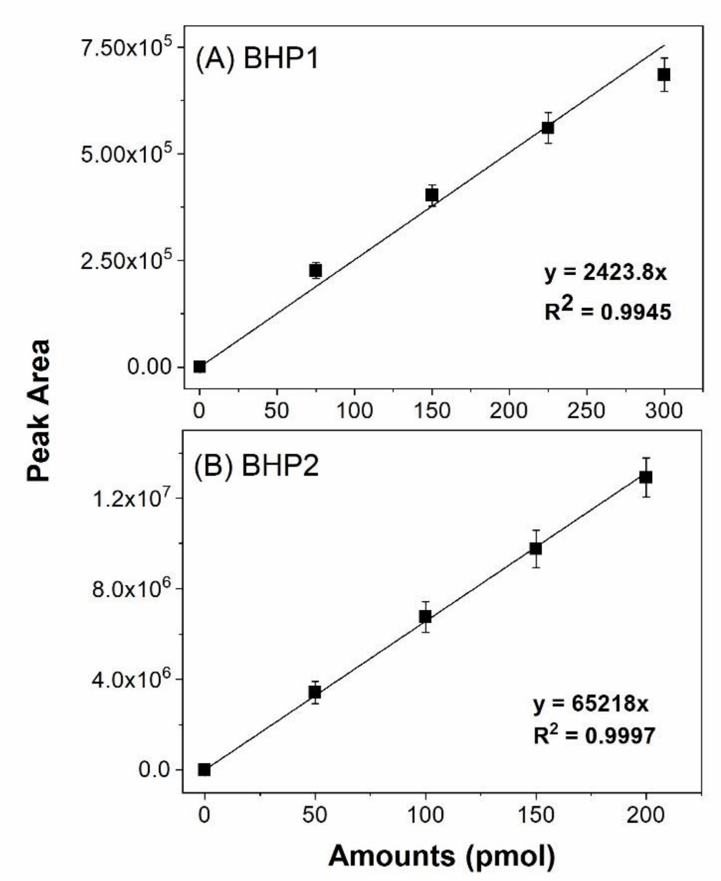

Figure S9. The standard curves of (A) BHP1 and (B) BHP2 for quantitation of BHP1 and BHP2.
